# The Contribution of Oculomotor Functions to Rates of Visual Information Processing in Younger and Older Adults

**DOI:** 10.1038/s41598-020-66773-5

**Published:** 2020-06-23

**Authors:** Deena Ebaid, Sheila G. Crewther

**Affiliations:** Department of Psychology and Counselling, School of Psychology and Public Health, College of Science, Health and Engineering, La Trobe University, Melbourne, VIC Australia

**Keywords:** Neuroscience, Cognitive ageing

## Abstract

Oculomotor functions are established surrogate measures of visual attention shifting and rate of information processing, however, the temporal characteristics of saccades and fixations have seldom been compared in healthy educated samples of younger and older adults. Thus, the current study aimed to compare duration of eye movement components in younger (18–25 years) and older (50–81 years) adults during text reading and during object/alphanumeric Rapid Automatic Naming (RAN) tasks. The current study also aimed to examine the contribution of oculomotor functions to threshold time needed for accurate performance on visually-driven cognitive tasks (Inspection Time [IT] and Change Detection [CD]). Results showed that younger adults fixated on individual stimuli for significantly longer than the older participants, while older adults demonstrated significantly longer saccade durations than the younger group. Results also demonstrated that older adults required longer threshold durations (i.e., performed slower) on the visually-driven cognitive tasks, however, the age-group time difference on the CD task was eradicated when the effects of saccade duration were covaried. Thus, these results suggest that age-related cognitive decline is also related to increased duration of saccades and hence, highlights the need to dissociate the age-related motor constraints on the temporal aspects of oculomotor function from visuo-cognitive speed of processing.

## Introduction

Most human behaviour is visually driven^1^, with visual attention considered the key driver of most perceptual, cognitive, and behavioural processes^2–4^. Vision and visual attention are known to undergo profound sensory and perceptual changes across the lifespan^5,6^, and have been a focus of much experimental research in the last 25 years^7–10^. These changes parallel those seen in several cognitive domains including attention, speed of processing, working memory, as well as motor slowing across the lifespan^10–13^. Despite this, debate still exists as to which domains are most susceptible to decline across the lifespan, and which may remain intact^13–19^.

Age related cognitive decline is commonly attributed to the anatomical and physiological impairments that occur in the eye, and central nervous system with advancing years^20–23^. Indeed, declines in normal age-related sensory function i.e., vision^6,23^ and audition^14,24–26^ have been shown to contribute significantly to the changes in cognitive ability seen across the lifespan. Such evidence underlies fundamental theories of cognitive aging, including the *Sensory Deprivation Hypothesis, the Common-Cause Hypothesis*, and *the Information Degradation Hypothesis*, which collectively suggest a strong interaction between declines in sensory system integrity with age and declines in cognitive abilities^5,27–29^ (See Ebaid and Crewther^30^ for a review on theories of cognitive aging). Most recently, age related physiological and behavioural changes to the visual system have been demonstrated using both flicker fusion thresholds as a critical measure of the fastest conducting Magnocellular (M) pathway function, and multifocal Visually Evoked Potentials (mfVEPS) that have demonstrated M and Parvocellular (P) pathway latencies increase with age, though the M generated peak latency increases are greater than those associated with the P pathway^23^. The M pathway is known to drive attention^31^ and eye movements, and so increased age related conduction latency and age related motor decline would be expected to influence eye movements associated with activation and shifts in attention^6,32^ in older individuals. Indeed, oculomotor functions including saccadic eye movements and fixations are surrogate measures of attention shifting^33,34^ that can provide insight into other complex cognitive abilities such as time taken to process visual information^35^. The link between attention and saccadic eye movements has been well demonstrated both with single cell electrophysiological measures of arousal in primates^33^ and in human fMRI and cortical stimulation research^33,34,36–39^. However, to date, there has been little consideration of the contribution of temporal aspects of oculomotor functions to cognitive decline with age.

Oculomotor functions are known to undergo functional decline across the lifespan in the absence of neurological impairment, with older adults often showing increased saccade latencies, decreases in peak velocity, and in some cases decreases in accuracy, particularly for volitional saccades^40–43^. Past research has demonstrated a strong age-related effect on saccade reaction time, indicating that young children aged between 5–8 years and elderly adults between 60–79 years showed similar slower time to onset saccades than young adults aged between 20–30 years^40^. Visual tracking performance (positional precision and smooth pursuit velocity gain of visual tracking) has shown similar age related effects^44^, while anti-saccade paradigms (saccade in the opposite direction of a visual target) have demonstrated both reduced inhibitory control and reduced motor speed^43,45^, which is in line with theories of cognitive aging including the *Inhibitory Deficit Hypothesis*^46^. This hypothesis suggests that older adults are more susceptible to task-irrelevant stimuli and have greater difficulty inhibiting distractions, resulting in heightened distractibility, reduced working memory capacity, and greater proactive interference^46^. To date however, no single experimental study has examined the effects of oculomotor function and visual attention on visuo-cognitive reaction time performance^47,48^ using eye tracking in healthy younger and older populations. For example, it is still unknown whether longer saccade durations contribute to the slowing of cognitive processing during visuo-cognitive tasks between younger and older adults, and if so, whether these differences in cognitive speed will still be apparent after controlling for the effect of saccade duration.

Thus, the current study aimed to examine eye movement patterns while text reading and during a Rapid Automatic Naming (RAN) task^49–51^ requiring participants to rapidly and accurately name a series of common objects or alphanumeric symbols. The current study also aimed to examine age-group differences in cognitive speed of processing on both simple and more complex visuo-cognitive tasks to determine whether older adults would again demonstrate a slowing of perceptual speed of processing, as per our previous study^19^. We then aimed to explore whether oculomotor function contributes differentially to observed cognitive speed of both age groups, and whether visuo-cognitive speed can predict performance on the RAN and reading tasks. The RAN task was chosen as it allows for assessment of temporal and spatial ocular functions while individuals engage in a relatively simple/automatic task (i.e., the naming of familiar stimuli). In addition, the reading task was chosen as an ecologically valid task of visual perception and attentional processing^19^. The Inspection Time (IT^52^); and Change Detection (CD^53^); tasks were selected as visuo-cognitive measures of threshold time required to process visual information with no reliance on hand motor-speed^19^, but with differential demands on eye movement patterns to encode the stimuli. Indeed, the IT task requires minimal saccadic movement to fixate the single centrally presented object, whereas the CD requires fixation and embedding in short-term memory of each of the four images in the array for successful detection of any change between two presentations. Hence, we expected that in contrast to the IT task, the CD task would induce more saccadic eye movements while accurately embedding the two visual arrays into memory.

Based on our previous study^19^, it was hypothesized that older adults would read the passage as fast as younger adults, but that older adults would name less stimuli during the objects and alphanumeric conditions of the RAN. We also expected older adults to perform slower on the IT and CD tasks compared to younger adults, and predicted that faster performance on these tasks would correlate with higher RAN scores, faster text-reading and better integrated oculomotor processing. Additionally, on the basis of confounding motor speed^13^ and previous oculomotor research in aging^40–43^, it was hypothesized that older adults would make more fixations, show increased fixation duration, and longer saccade durations during all the RAN and reading tasks compared to younger adults. In regard to statistically controlling the effect of oculomotor function from performance on visuo-cognitive tasks, we predicted that doing so would decrease the age-group variance seen on the CD, but not on the IT, due to the CD tasks requirement for rapid eye movements. It was also hypothesized that performance on the visuo-cognitive tasks (IT and CD) would significantly predict RAN naming and reading scores, given that the IT and CD are widely accepted measures of visual processing speed and visual short-term memory capacity^10,54^.

## Results

### Descriptive statistics and age-group differences for IT, CD, RAN, text-reading and oculomotor functions

Descriptive statistics and age-group differences for scores on the IT, CD, RAN (naming score), text-passage (reading score) and oculomotor measures (number of visual fixations, fixation duration and saccade duration) during the RAN and reading tasks are presented in Table 1.

**Table 1:** Descriptive Statistics and Age-Group Differences for Scores on the IT, CD, RAN, Text-Reading Tasks and Oculomotor Function for Younger and Older Adults.

Measure	Younger Adults				Older Adults				*Age-Group Differences*	
	*N*	Range	*M*	*SD*	*N*	Range	*M*	*SD*	*p (*2 *-tailed)*	*η*^*2*^
**IT (ms)**	45	20.00–200.00	53.26	31.51	22	34.90–232.00	111.04	60.25	0.000**	0.287
**CD (ms)**	45	110.00–1310.00	637.40	299.52	22	42.00–1798.00	839.64	494.39	0.041*	0.062
**RANObjects** (naming score)	45	63.00–127.00	89.67	17.25	22	59.00–112.00	79.50	14.536	0.020*	0.080
No. Fixations	45	88.0–150.0	120.47	14.012	22	113.0–152.0	127.00	10.52	0.058	0.054
FixationDur (ms)	45	297.89–574.03	427.85	63.03	22	310.00–466.20	379.26	47.94	0.002**	0.135
SaccadeDur (ms)	45	44.50–169.15	76.24	28.53	22	37.80–207.00	95.59	49.65	0.043*	0.062
**RANLetters**(naming score)	45	100.0–338.0	163.689	45.48	17	105.00–304.00	180.88	42.93	0.183	0.029
No. Fixations	45	114.0–201.0	165.47	17.01	17	151.00–209.00	173.24	42.97	0.099	0.045
FixationDur (ms)	45	251.00–454.00	314.94	39.74	17	233.30–350.00	283.88	31.76	0.005**	0.122
SaccadeDur (ms)	45	29.00–134.00	49.24	17.98	17	37.00–112.00	62.92	22.27	0.015*	0.094
**RANNumbers**(naming score)	45	90.00–293.00	163.89	42.46	22	130.00–225.00	175.86	28.87	0.237	0.021
No. Fixations	45	126.0–204.0	168.89	15.18	22	134.00–204.00	177.09	16.95	0.050	0.058
FixationDur (ms)	45	240.40–399.00	301.61	33.86	22	237.00–329.00	277.40	23.45	0.004**	0.123
SaccadeDur (ms)	45	33.00–150.00	55.56	22.39	22	36.00–189.00	70.59	41.58	0.058	0.054
**Passage Reading Score** (ms per word)	45	175.51–428.57	301.27	2.40	14	210.00–367.35	301.47	2.57	0.990	0.000
No. Fixations	45	25.00–51.00	38.82	5.67	14	31.00–108.00	43.14	19.30	0.183	0.031
FixationDur (ms)	45	264.20–390.80	329.77	27.63	14	279.30–427.90	325.64	41.61	0.668	0.003
SaccadeDur (ms)	45	4.60–142.20	46.93	27.88	14	17.00–135.60	57.04	29.60	0.248	0.023

*Note:* Naming score = number of stimuli named on the RAN in 60 seconds, No. Fixations = number of fixations made during the RAN tasks and during the text-reading passage, Fixation Duration = Mean fixation time in milliseconds (ms) per stimuli/word during the RAN and reading tasks, Saccade Duration = Saccade duration from one fixation point to the next during RAN and reading tasks, Passage Reading score = time taken (ms) to read each word in the text-passage.

### Age-group differences in performance on the IT, CD, RAN, text-reading and oculomotor functions

**Figure 1:**
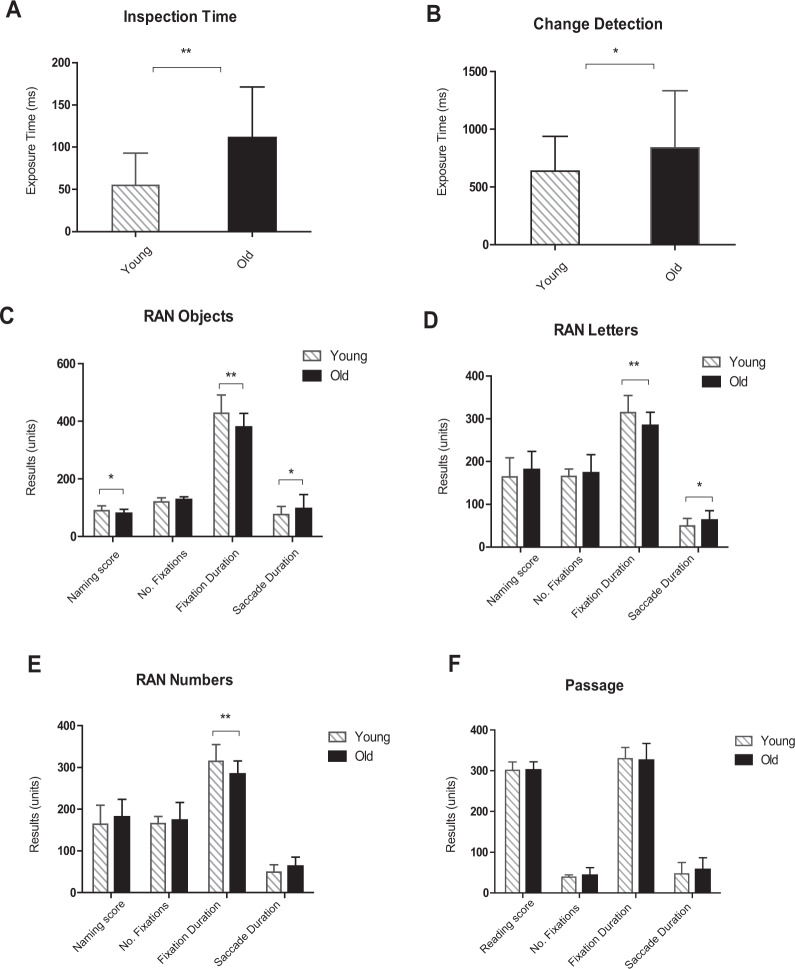
(**A–F**) Age-group differences in performance on the IT, CD, RAN objects/letters/numbers and text-passage. *Note*: Naming score = number of stimuli named on the RAN in 60 seconds, No. Fixations = number of fixations made during the RAN tasks and during the text-reading passage, Fixation Duration = Mean fixation time in milliseconds (ms) per stimuli/word during the RAN and reading tasks, Saccade Duration = Saccade duration from one fixation point to the next during RAN and reading tasks, Passage Reading score = time taken (ms) to read each word in the text-passage.

An independent samples t-test was performed to determine whether there were differences in RAN naming score, text-reading score, oculomotor function, or the IT and CD tasks between young and older adults. Significant age-group differences were demonstrated for the IT task (*t* (65) = −5.109, *p* < .001, *η*^*2*^ = 0.287), as well as for the CD task (*t* (65) = −2.080, *p* < .05, *η*^2^ *=* 0.062) where younger adults required a significantly shorter threshold exposure duration to identify a familiar visual stimulus or detect change between two visual arrays, respectively. In regard to ocular functions, fixation and saccade duration during RAN tasks were the predominant differences between groups. Specifically, older adults demonstrated significantly longer saccade durations, while younger adults fixated on individual stimuli for significantly longer in the RAN objects and alphanumeric conditions, but not during the reading task. These results are depicted in Fig. 1A–F.

### Age-group differences in performance on the IT and CD while controlling for saccade duration

A Multivariate Analysis of Covariance (MANCOVA) was conducted to examine group differences in time required to process visual information and detect change between two visual arrays on the IT and CD, respectively, while controlling for saccade duration. Preliminary assumption testing was conducted to check for normality, linearity, homogeneity of variance and multicollinearity, with no violations noted. Saccade duration during the reading task was chosen as the covariate given that it can be considered the most automatic and ecologically valid cognitive task with the least cognitive load^55^. After controlling for saccade duration, results still demonstrated a significant large effect of age-group on time required to process visual information on the IT, *F*(2, 57) = 13.713, *p* < 0.001, *η*^2^ = 0.329. Further, after controlling for saccade duration, age-group explained 32.90% of the variance in IT scores. Results also indicated that after controlling for saccade duration, there was no longer a significant age group difference in time to detect change between two visual arrays on the CD, *F* (2, 57) = 2.214, *p* = 0.119, *η*^2^ = 0.073, whereby only 7.3% of the variance in CD scores were explained by age-group. Results of the MANCOVA are presented in Table 2.

**Table 2:** Multivariate Analysis of Covariance (MANCOVA) between Age-Groups and Performance on the IT and CD while Controlling for Saccade Duration (ms).

Variable	Sum of Squares	*df*	Mean Square	*F*	*p*	Partial *η*^2^
IT	50.038	57	25.019	13.713	0.000	0.329
CD	601.36	57	300.681	2.214	0.119	0.073

### Relationships among IT, CD, RAN, text-reading and oculomotor functions

#### Pearson’s correlation

**Figure 2:**
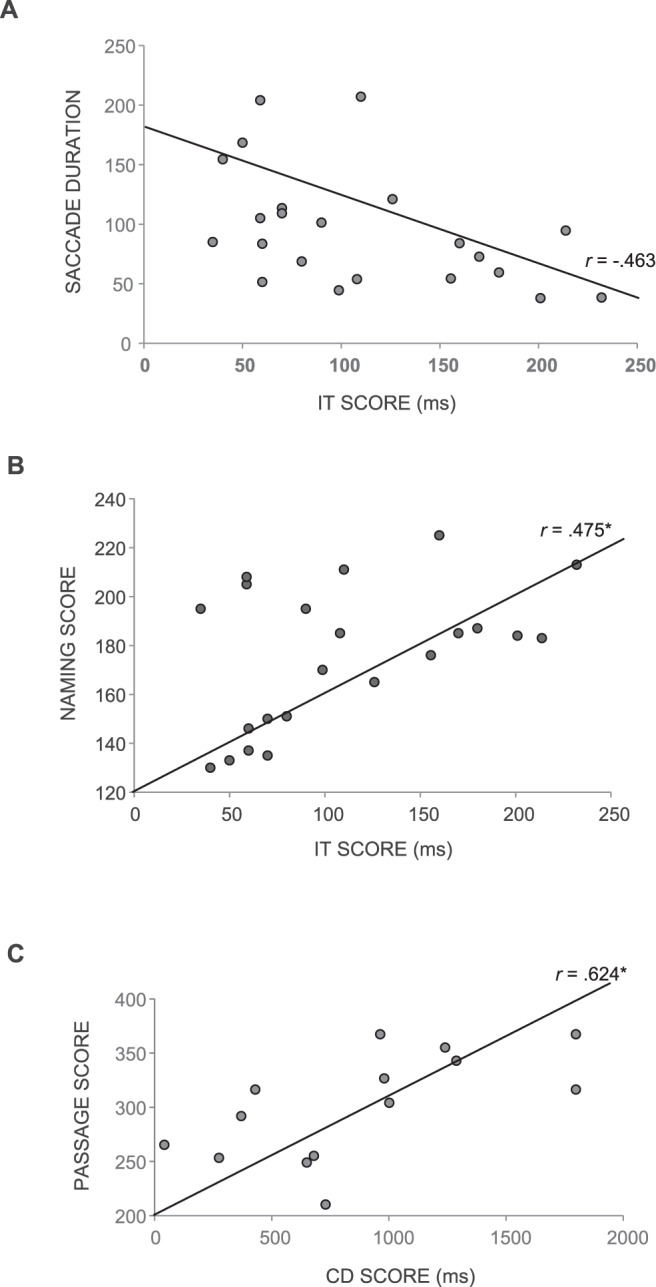
(**A**) Scatter plot depicting the correlation between Inspection Time (IT) score and average saccade duration during the RAN Objects task for older adults. *Note*, faster/better performance on the IT is indicated by a shorter IT score. (**B**) Scatterplot depicting the correlation between Inspection Time (IT) score and the naming score during the RAN Numbers for older adults. *Note*, faster/better performance on the IT is indicated by a shorter IT score. (**C**) Scatterplot depicting the correlation between Change Detection (CD) score and the text-passage (ms per word) for older adults. *Note*, faster/better performance on the CD is indicated by a shorter CD score.

To investigate the strength, direction and significance of associations between cognitive performance on the IT, CD, RAN, reading and oculomotor function, correlational analyses using Pearson’s Correlation were performed separately for younger and older adults. No significant correlations were revealed for the younger adults. For the older adults, results revealed a significant negative correlation between IT score and saccade duration during the RAN Objects task (*r* = −0.463, *p* < 0.05) indicating that shorter threshold exposure duration to correctly identify a visual stimulus was associated with significantly longer saccade durations. Additionally, results demonstrated a significant positive correlation between IT score and RAN Numbers naming score for older adults (*r* = 0.475, *p* < 0.05), indicating that a shorter threshold exposure duration was significantly associated with naming less numbers. Furthermore, there was a significant positive correlation between CD score and the text passage for older adults, indicating that shorter threshold exposure duration to correctly identify change between two visual arrays was associated with less time required to read each word in the text-passage. A correlation table of IT and CD with RAN and reading tasks as well as oculomotor measures is shown in Table 3. Significant results are also depicted in Fig. 2A–C.

**Table 3:** Pearson’s Correlations (r) between Inspection Time (IT), Change Detection (CD), RAN, and Three Measures of Ocular Functions in Younger and Older Adults.

	Younger Adults		
	*N*	IT	CD
RANObjects	45	−0.041	−0.001
No. Fixations	45	−0.100	−0.162
FixationDur	45	0.181	0.111
SaccadeDur	45	−0.163	0.072
RANLetters	45	−0.095	−0.125
No. Fixations	45	0.149	0.106
FixationDur	45	0.201	−0.213
SaccadeDur	45	0.092	0.255
RANNum	45	−0.024	−0.061
No. Fixations	45	0.195	0.208
FixationDur	45	−0.131	−0.214
SaccadeDur	45	−0.085	0.011
Passage	45	−0.172	0.238
No. Fixations	45	−0.167	0.262
FixationDur	45	−0.203	−0.028
SaccadeDur	45	−0.012	−0.056
	**Older Adults**		
	***N***	**IT**	**CD**
RANObjects	22	0.187	−0.036
No. Fixations	22	0.352	−0.081
FixationDur	22	0.204	−0.050
SaccadeDur	22	−0.463*	0.107
RANLetters	17	0.200	−0.293
No. Fixations	17	0.006	0.129
FixationDur	17	−0.164	−0.382
SaccadeDur	17	0.058	0.309
RANNum	22	0.475*	−0.323
No. Fixations	22	0.193	−0.060
FixationDur	22	−0.125	0.209
SaccadeDur	22	−0.076	−0.262
Passage	14	−0.354	0.624*
No. Fixations	14	0.095	−0.006
FixationDur	14	0.088	−0.461
SaccadeDur	14	−0.377	0.101

*Note*. Significant values are presented in bold.

#### Hierarchical multiple regression

Hierarchical multiple regression was used to assess the ability of visual processing speed (using IT and CD scores) to predict naming scores for the three RAN tasks (Objects, Letters, Numbers) as well as the passage reading score after adjusting for age. Given that IT and CD represent widely accepted measures of visual processing speed and visual short-term memory capacity^10,54^, they were chosen as the most appropriate measures to predict performance on the RAN and reading tasks. Preliminary analyses were conducted to ensure no violation of the assumptions of normality, linearity, multicollinearity and homoscedasticity, with no violations noted. For all analyses, age was entered at step 1, followed by IT and CD score at step 2.

### RAN Objects

Age significantly explained 9.10% of the variance in rapid naming of objects, *R*^2^ = 0.091, *F* (1, 66) = 6.525, *p* = 0.013. After the entry of IT and CD scores at step 2, IT and CD scores explained an additional 0.04% of the variance in RAN Objects naming score, and this was not a statistically significant contribution (*p* = 0.881). Results demonstrated that the model as a whole which explained 9.5% of the variance in RAN Object naming scores was not statistically significant, *F *(2, 66) = 2.20, *p* = 0.097.

### RAN Letters

Age explained 2.8% of the variance in letter naming scores, and this was not statistically significant, *R*^2^ = 0.028, *F*(1, 60) = 1.784, *p* = 0.191. At step 2, IT and CD scores explained an additional 2.8% of the variance in naming scores, though this was not statistically significant, *R*^2^ = 0.056, *F* (2, 58) = 1.147, *p* = 0.338. Results also demonstrated that the model as a whole, which explained 5.6% of the variance in letter naming scores was not statistically significant (*p* = 0.338).

### RAN Numbers

At step 1, age did not significantly explain the variance in number naming scores, *R*^2^ = 0.022, *F *(1, 65) = 148, *p* = 0.227. The entry of IT and CD scores at step 2 predicted an additional 3.3% of the variance in RAN Numbers naming score, though this was not a statistically significant contribution, *R*^2^ = 0.055, *F *(2, 63) = 1.421, *p* = 0.342. Results also showed that the model as a whole, which predicted 5.50% of the variance in RAN Numbers naming score was not statistically significant (*p* = 0.308).

### Text-Passage

At step 1, age explained 0.1% of the variance in text-passage reading score, and this was not statistically significant, *R*^2^ = 0.001, *F* (1, 57) = 0.077, *p* = 0.782. The entry of IT and CD scores at step 2 predicted an additional 14.80% of the variance, and this was a statistically significant contribution, *R*^2^ = 0.149, *F *(2, 55) = 3.215, *p* = 0.012. Results also showed that the model as a whole, which predicted 14.90% of the variance in text-passage reading scores, was statistically significant (*p* = 0.030).

R^2^ change (*ΔR*^2^) and semi-partial correlation coefficients *(sr)* across all analyses are presented in Table 4.

**Table 4:** Hierarchical Multiple Regressions of Age, IT, and CD Predicting Naming Score on RAN Tasks and Text-Passage Reading Score.

Predictors	RAN Objects		RAN Letters		RAN Numbers		Text-Passage	
	*ΔR*^2^	*sr*	*ΔR*^2^	*sr*	*ΔR*^2^	*sr*	*ΔR*^2^	*sr*
Step 1	0.091	−0.302	0.028	0.168	0.022	0.149	0.001	−0.037
Age								
Step 2	0.004		0.028		0.033		0.148	
Age		−0.275		0.171		0.080		−0.007
IT score		0.059		−0.005		0.121		−0.152
CD score		−0.003		−0.164		−0.109		0.331

*Note*. Four separate hierarchical regressions were conducted for Age, IT and CD predicting naming score on the RAN Objects, Letters, Numbers and the text-passage, respectively.

### Factor analysis

Given the number of variables in the current study, the factorability of all 19 variables was examined and subjected to Principal Components Analysis (PCA) as a means of data reduction. The suitability of the data for factor analysis was assessed prior to performing PCA. Inspection of the correlation matrix revealed the presence of several coefficients of 0.3 and above. The Kaiser-Meyer-Olkin value of 0.50 was slightly below the recommended value of 0.60^56^, though the Bartlett’s Test of Sphericity^57^ reached statistical significance, supporting the factorability of the correlation matrix.

PCA revealed the presence of eight components with eigenvalues exceeding 1, explaining 21.41%, 12.92%, 11.53%, 9.46%, 6.97%, 6.34%, 5.89%, 5.42% of the variance, respectively. An inspection of the scree plot revealed a clear break after the second component, and thus it was decided to retain the two greatest components of variance for further analysis.

The two-component solution explained a total of 34.33% of variance, with component 1 contributing 21.41% and component 2 explaining 12.92% of the variance. Oblimin rotation was performed to aid the interpretation of these two components. The rotated solution revealed the presence of simple structure^58^, with both components showing several moderate to strong loadings and all variables loading substantially on only one component. Table 5 depicts these results.

**Table 5:** Pattern and Structure Matrix for PCA with Oblimin Rotation for the 19 Dependent Variables.

Variable	Pattern Coefficients		Structure Coefficients		Communalities
	1	2	1	2	0.529
SaccadeDur (RAN Numbers)	0.701		0.694		0.302
FixationDur (RAN Objects)	−0.672		−0.678		0.225
Naming Score (RAN Numbers)	0.631		0.631		0.150
Naming Score (RAN Letters)	0.613		0.611		0.192
SaccadeDur (Passage)	0.599		0.605		0.474
SaccadeDur (RAN Objects)	0.583		0.580		0.340
FixationDur (Passage)	−0.521		0.536	0.323	0.374
SaccadeDur (RAN Letters)	0.520		−0.523		0.266
No. Fixations (RAN Objects)	0.390		0.399		0.452
Passage Score (ms per word)	−0.349		−0.341		0.374
No. Fixations (Passage)	−0.343		−0.330		0.399
Age		0.681		0.693	0.457
No. Fixations (RAN Numbers)		0.668		0.660	0.523
FixationDur (RAN Letters)		−0.609		−0.623	0.499
FixationDur (RAN Numbers)	−0.406	−0.577	−0.437	−0.598	0.141
IT score		0.536		0.541	0.172
No. Fixations (RAN Letters)		0.514		0.510	0.275
CD score		0.474		0.472	0.379
Naming Score (RAN Objects)	−0.521	−0.333	0.694	−0.321	0.529

*Note*. Factor loadings <0.3 are suppressed.

## Discussion

The aim of the current study was to examine and compare the temporal characteristics of ocular functions (saccades and fixations) during rapid automatized naming and text reading in a healthy educated sample of young and older adults, and to investigate whether such functions contribute to threshold times on visuo-cognitive tasks varying in complexity. The current study also aimed to explore the predictive validity of the IT and CD tasks for rapid automatized naming and text-reading. The key finding was that cognitive speed on the visuo-cognitive tasks was slower for older adults, and that saccade durations during the RAN conditions were longer for older adults, though the age-group effects on the visuo-cognitive tasks were reduced when saccade duration was controlled. Specifically, after controlling for the effects of saccade duration, age-group differences in performance on the four-object array CD task were no longer present, though age-group differences on the single stimulus IT task remained significant. Furthermore, older adults showed a slower naming speed for the objects condition of the RAN but not for the more automatic number or letter conditions, thus partially supporting hypotheses that older adults would name less stimuli during the RAN tasks. Again supporting hypotheses, older adults demonstrated comparable reading speed to younger adults during the text-passage task. The demonstration of only three significant relationships between visuo-cognitive measures (IT and CD), RAN and reading tasks, as well as ocular measures, did not fully support the hypothesis that IT and CD performance would significantly correlate with faster RAN/reading and better integrated eye movements. Similarly, results from the regression analysis only demonstrated significant predictive validity of the IT and CD measures for performance on the reading task, and so also only provides partial support for the hypothesis that IT and CD would significantly predict performance on the naming and reading tasks. Indeed, the IT and CD represent established measures of visual processing speed and visual short-term memory capacity, and have been considered predictors of other more complex cognitive tasks^10,54^. Despite this, these tasks have not often been considered in terms of the requirement for attention shifting and the relation to aspects of eye movements.

### Threshold exposure durations on the Inspection Time (IT) and Change Detection (CD) tasks in young and older adults

In line with hypotheses, older adults performed significantly slower i.e., required longer exposure duration to detect a familiar visual stimulus and to identify change between two visual arrays as indicated by performance times on the IT and CD, respectively. These results are consistent with the past research reporting on declines in visuo-cognitive processing speed in healthy aging^12,23,59–62^. Findings from the current study are also in line with theories of aging which postulate that sensory decline has an indirect influence on cognitive performance i.e., the *Sensory Deprivation Hypothesis, the Common-Cause Hypothesis*, and *the Information Degradation Hypothesis*^5,27–29^, and with theories of cognitive slowing with age, i.e., *the Processing Speed Theory*^12^. Given the age of our older population, it may be the case for example, that a lack of optimized sensory input over a prolonged period of time (i.e., due to natural declines in vision and audition) has resulted in slower precortical processing^23^ and potentially associated neural atrophy^5,27,28^. These factors could in turn impede speed of cognitive performance as seen in the current study. This explanation is in line with the fundamental premise of the *Sensory Deprivation Hypothesis*^29^.

As expected, when saccade duration was accounted for prior to assessing age-group differences, there was no longer a significant difference in cognitive processing speed between younger and older adults as measured by the visuo-cognitive CD task. This is likely due to increased saccade durations of the older group affecting efficient attentional processing of the four stimuli, as well as time to embed the array in short term memory, resulting in an increased threshold duration required to detect change between the two visual arrays. Certainly, rapid eye movements and efficient scanning of the alphabetic stimuli in the two arrays of the CD task are necessary for accurate performance on this task. On the other hand, even when saccade duration was covaried, age-group differences remained on the IT task, where minimal saccadic eye movements or embedding into short-term memory are required to identify a single stationary object. This finding was in line with our hypothesis and is also consistent with those found by Brown *et al*.^23^ who tested both flicker fusion thresholds and latency of the two retinal pathways with mfVEPS and showed that the M generated peak latency increases are greater than associated P changes in healthy older adults. These results also add a new dimension into influential theories of cognitive aging which typically suggest a generalized slowing and inhibition in cognitive processing i.e., Salthouse^12^,Hasher and Zacks^46^, without accounting for visual processing and oculomotor function.

### Oculomotor functions in young and older adults

The number of visual fixations for the objects, alphanumeric and the text passage conditions were not significantly different between age groups, though there was a trend for older adults to make more fixations than younger adults across all tasks. Additionally, fixation duration was significantly longer in younger adults compared to older adults for the objects and alphanumeric conditions of the RAN but not for the text-passage, which may suggest that younger adults required longer duration to encode and access the name of single discrete stimuli in these conditions. Alternatively, this may infer a more efficient oculomotor strategy used by younger adults, by fixating for a longer duration on the target stimuli while inhibiting distractors (i.e., the surrounding objects^43^), enabling more accurate encoding of the target stimuli. Despite some evidence in favor of this suggestion, many lines of research report that longer fixations are associated with less efficient processing in children^61,62^ and older adults^43,45^, i.e., longer fixation times are associated with slower reading rates^63^. This finding was contrary to our hypotheses, given that we have previously demonstrated that a group of healthy younger adults required shorter presentation time (i.e., 49 ms compared to 136 ms for older adults) to identify a familiar single stationary visual stimulus^19^, though eye-tracking was not available or utilized in our previous study. The current findings also contradict previous research which has interpreted longer fixations to be reflective of the individual requiring more time to acquire visual and orthographic information from the stimuli in preparation for the correct response^64,65^. However, it is important to note that the older adults in the current study showed longer threshold exposure times to complete the IT and CD tasks, while demonstrating comparable performance to the younger adults on the reading and alphanumeric conditions of the RAN. Given this, it is unlikely that the longer fixation durations shown by the younger adults are reflective of inefficient visual encoding/requiring longer to embed stimuli into memory as previously suggested^64^. Indeed, as alluded to earlier, it may be the case that a different cognitive strategy was used by the younger individuals during the naming tasks, which did not impact on their behavioral performance^66^ (i.e., naming and reading scores were comparable across both groups). However, the precise cognitive strategy used by the two groups remains a subject of debate.

Saccade duration for older adults was significantly longer in the Objects and Letters condition of the RAN compared to younger adults. Though age-group differences in saccade duration during the RAN Numbers condition did not reach statistical significance, older adults still demonstrated a longer saccade duration compared to younger adults. These findings are in line with past research^67^ and presumably associated with slower motor movements even in healthy aging^13^. This finding was consistent with our predictions and previous literature^23,68^ indicating age related vulnerability associated with neural control mechanisms for the direction and amplitude of saccadic eye movements, that are in part controlled by the basal ganglia^68^, the motor neurons of the oculomotor nuclei^69^, and the parietal cortex. Such regions are critical in the interface between attention and motor planning, including saccadic eye movements^70^. Again, an alternative explanation for the different temporal trajectories of the age-related gaze patterns in facilitating similar naming scores in the alphanumeric conditions of the RAN, could be related to time needed to embed visual stimuli in memory, and may also point to different cognitive strategies used by the two age groups, though what specific strategy is unclear.

### Rapid Automatic Naming (RAN) performance for young and older adults

Our prediction that older adults would name less stimuli on the common-objects and alphanumeric conditions of the RAN was partially supported, with older adults naming significantly fewer stimuli during the RAN Objects condition compared to younger adults. Interestingly however, in the Letters and Numbers conditions, older adults on average named more alphanumeric stimuli compared to younger adults, though these differences did not reach statistical significance. With reference to older adults performing slower on the RAN Objects, this condition of the task requires greater attention to individual objects that are presumably more variable and less familiar/predictable despite the names having been practiced before the experiment. Moreover, it may be the case that naming the object stimuli was less automatic than rapidly naming numbers and letters which have no alternative names^12,71–73^. Hence, naming alphanumeric symbols is likely to require less sustained attention, less neural resources and less cognitive processing time to successfully verbalize the nominated stimulus^15^. By comparison, object naming often requires more conscious effort, especially if older adults were required to inhibit a name that they would preferentially use for a particular object instead of the experimenter prescribed name. The inhibition of an automatic response may have impaired overall task performance, as explained by the *Inhibitory Deficit Hypothesis*^46^. These findings are also consistent with suggestions made by Madden^15^ who proposed that older adults perform as fast and as accurately on visual search tasks which require top-down processing (i.e., the alphanumeric conditions of the RAN), but perform slower in tasks that require inhibition of distracters i.e., potentially the case in the objects condition.

It should also be noted that differences in naming speed between younger and older adults may be due to sex-differences within the two age-groups. More specifically, females represented approximately 87% of the younger adults, and 63% of the older adults and thus, the overrepresentation of females in the younger group may have contributed to faster naming speed in the RAN Objects condition. Indeed, in a review investigating gender differences in processing speed^74^, it was reported that females demonstrate a faster processing speed on tasks involving digits, alphabets and rapid naming, while men are typically faster on finger-tap reaction time tests^74^.

### Correlations between visual processing speed, naming speed and oculomotor performance in young and older adults

Correlation analyses did not demonstrate any significant relationships between IT and CD performance and oculomotor measures during the RAN or reading tasks for younger adults. For the older adults however, results demonstrated a significant negative correlation between the IT score and mean saccade duration during the RAN Objects condition, indicating that where shorter threshold exposure duration was required to correctly identify a visual stimulus, then it was significantly associated with longer saccade durations. This finding contradicted our initial prediction that faster IT threshold performance would correlate with shorter saccade durations (i.e., *faster* saccades) and may indeed be one of the age-related strategies adopted by well-educated aged readers. Indeed Miyata, *et al*.^75^, have demonstrated that faster reading is associated with larger horizontal saccadic movements, as well as shorter fixation times when measured while Japanese participants read novels in their own language. However, Miyata, *et al*.^75^ suggested that larger variances in ‘saccade sizes’ may be due to the use of irregular or unsteady eye-movement strategies, which may apply to the older adults in the current study when verbalizing less familiar object names. It may also be the case that there was a trade-off between faster visual processing and saccade durations for older adults while performing the RAN Objects task due to potential degeneration in the neural structures associated with visuomotor processing^40–43,68^.

Although correlation analyses in the current study also revealed a significant positive correlation between IT threshold exposure duration and RAN Numbers naming score for older adults, this did not entirely support our hypothesis and highlighted the fact that the ‘number of items named in 60 seconds’ may not be entirely reflective of efficient visual processing alone. Rather, it is important to note that the RAN tasks require rapid scanning of a visual scene, several ocular processes such as accommodation and focus, encoding and embedding the stimuli into memory, access to vocabulary, and orofacial motor movements in order to name the stimuli. Results from the current study also demonstrated a significant positive correlation between CD score and the text passage score for older adults, indicating that shorter threshold exposure duration to correctly identify change between two visual arrays was associated with less time required to read each word in the text-passage. This finding supported the hypothesis that faster visuo-cognitive speed would correlate with more efficient eye movements, as demonstrated during the text-passage for older adults. This finding is in line with previous work reporting that reading is largely reliant on rapid visual processing, visual attention, and continuous inhibition of distractors, in addition to continuous access to lexical storage, integration of sublexical, orthographic, phonological, and lexico-semantic information and working memory^76,77^. Though this is typically the case for both young and older adults, no significant correlations between the visuo-cognitive tasks (IT and CD) and oculomotor variables during the reading task were demonstrated for the younger sample.

The lack of any additional significant correlations between IT, CD and oculomotor measures in the younger group in the current study is similar to findings reported by Garaas and Pomplun^78^, who also used a single object Inspection Time task alongside oculomotor measures (fixation duration and saccade latency) in a sample of young to middle-aged adults. It should be noted that our task only aimed to measure minimum threshold required for visual identification of a single centrally placed object and hence, required minimal eye movements. This may explain why only a small number of significant correlations were demonstrated between the IT task and oculomotor measures. Furthermore, it may also be noteworthy that the average IT threshold durations demonstrated in the current study for both younger and older adults were well below typical saccade latencies, reported to be ~200 ms in laboratory settings^79,80^. Taken together, these findings emphasise that the simplicity of the more basic visual IT and CD tasks with their minimal requirement for eye movements or verbal motor responses, are unlikely to be reliably related to the more holistic cognitive task demands of RAN and text reading. Indeed, the RAN and text-reading tasks carry the requirement of object identification, access to lexicon, rapid verbalization of the words or objects, as well as organized sequential eye movement associated with shifts in attention.

### Visual processing speed predicting rapid naming and reading

In the current study, regression analyses demonstrated some predictive validity of IT and CD measures in predicting reading scores, but not in predicting RAN performance. More specifically, in relation to the text-passage task, the model which included age, the IT, and CD scores significantly predicted 14.90% of the variance in text-passage scores. This finding was in line with our predictions and past research as discussed previously, reporting that reading is heavily reliant on rapid processing of visual information and other complex cognitive processes such as visual attention, continuous inhibition of distractors and working memory^76,77^. This finding also supports our results from the correlation analysis demonstrating a significant association between CD score and text-passage score for older adults. In this model, age did not significantly predict scores on the text-passage task and only explained 0.1% of the variance, which is in line with comparable text-passage scores between age-groups. For the RAN Objects, age was the main contributor to scores (9.10%), with IT and CD only contributing an additional 0.04%. The model which included IT and CD scores while accounting for the effect of age, did not significantly predict any other RAN scores, and this was an unexpected finding which contradicted hypotheses.

### Exploration of the factorability of the measures

The factor analysis enabled exploration of the factorability of the variables in the current study. The results revealed a two-component solution which accounted for a total of 34.33% of variance. The first component predominantly included measures of saccade duration and naming scores on the RAN and passage tasks and contributed to 21.41% of the variance. Together, the variables within this component support theoretical explanations that rapid naming is simultaneously reliant on fast saccadic eye movements and coordinated vocalization of the series of common objects or alphanumeric symbols. Additionally, the variables in the first component of the factor analysis appear to represent the motor component of visual processing (i.e., saccades and orofacial movements required for verbalization of names). Indeed, in a study conducted by Gordon and Hoedemaker^81^,the authors used concurrent recordings of young adult participants’ vocalizations and eye movements during RAN tasks to examine how the coordination of visual and vocal processes impact RAN performance. The authors reported that good performance on the RAN requires rapid eye movements and slower articulation that is coordinated in time so that the eyes are sufficiently ahead of the voice, preparing for the upcoming RAN stimuli^81^.

The second component which predominantly included variables associated with fixations (i.e., fixation count and duration) as well as the IT and CD, is also reflective of theoretical understanding of oculomotor parameters in visual perception, indicating that visual fixations and duration are reflective of time required to acquire necessary visual information^82^. Similarly, the IT and CD tasks measured threshold time required for correct identification of a visual stimulus and decision on change/no change between two rapidly presented visual arrays. As alluded to earlier, the IT and CD tasks in the current study measured minimum threshold durations of a single centrally placed object, and change detection between two centrally placed visual arrays, respectively, with both tasks requiring minimal eye movements. Thus, it is likely that the items comprising component two of the factor analysis are reflective of more complex cognitive processes including perceptual speed, and time needed to encode a visual stimulus and embed into memory^64,65,83^.

### Limitations

The similarly educated individuals in our sample are both a strength and weakness of this study Similar population education and current interests in learning enabled robust comparison between groups, though limits the generalizability of the study findings to a more general, less educated population of both age groups. Our study is also limited in terms of the assessments of health status, in that we used self-report rather than neuropsychological assessments of affect or potential mild cognitive impairment (MCI)^84^ in either group. Thus, it is unclear whether age-related differences in the RAN Objects condition may be due to factors such as MCI in the older group^84^. However, the comparable scores between age groups on the alphanumeric RAN tests and text reading argues against MCI. Our uneven sample sizes between age groups and relatively small sample of older participants may have also decreased the power of some statistical analyses conducted, particularly those requiring a large sample size (i.e., factor analyses). Given this, future studies and analyses are likely to benefit from use of a larger sample of younger and older participants with various educational backgrounds when exploring similar research questions. Though sex differences in cognitive ability was not a focus of this study, future research may also benefit from having an even spread of genders within each age-group to examine whether gender may contribute to differences seen in cognitive and oculomotor ability.

### Conclusions & future directions

Our study is among the first to assess and compare robust cognitive measures of perceptual speed, as well as spatial and temporal aspects of gaze patterns during RAN tasks and text reading in healthy educated samples of younger and older adults. Our results show that oculomotor functions become slower with age and suggest use of different strategies that may further contribute to the slower cognitive processing on complex visuo-cognitive tasks seen across the lifespan. Interestingly, after covariation of saccade durations, the threshold exposure time needed to detect change was not significantly different between younger and older groups. To our knowledge, this study has been the first to examine the predictive validity of the IT and CD to performance on the naming and reading tasks.

Our quantification of the temporal aspects of gaze patterns during performance on visual tasks elucidates perspectives on the time taken to activate and deactivate saccades, demonstrating that older adults have longer saccade durations, though this does not always denote slower behavioral performance. Thus, we can conclude that the two age groups may utilize slightly different temporal strategies to achieve similar performance on tasks such as naming alphanumeric stimuli and reading. However, whether the age-group disparity reflect unique differences to activate attention and initiate saccades, motor speed of saccade, or cognitive aspects of the tasks, or whether the differences are reflective of all three components remains to be determined. It may be of interest for future research into rapid automatized naming and ocular performance in similarly educated younger and older adults to utilize a verbal intelligence measure such as the National Adult Reading Test (NART)^85^ in order to elucidate whether both groups demonstrate comparable performance.

Overall, our findings extend understanding of ocular function with age, and demonstrate that eye movements are a non-invasive and innovative measure of cognitive function which should continue to be employed in cognitive aging research, and potentially as clinical measures of cognitive processing. Future research should aim to examine whether oculomotor function during visual tasks are predictive of cognitive performance on other robust measures of cognitive speed with different task demands, as literature in this area remains relatively rare.

## Method

### Participants

This study included 67 healthy educated participants, some of whom which participated in a previous study conducted by our lab, and thus have been described in our previous work^19^. Participants were divided into a younger and older adult group, where the younger sample comprised of 45 adults aged between 18–25, and the older group included 22 healthy older adults aged between 50–81 years. The younger adults were first year Psychology students from La Trobe University, Melbourne, and received course credit for their participation, whereas the older cohort were recruited from the University of the Third Age (U3A) Manningham, and received a $20 Coles-Myer voucher for their participation^19^. U3A is an international volunteer organization where interested older individuals are able to come together and collaboratively learn, rather than for any qualifications (for more information please visit www.u3a.org.au). All participants had normal or corrected-to-normal visual acuity (6/6) and no history of ophthalmological disease. All experimental tasks were visually based and were all presented at suprathreshold visual contrast. A demographic questionnaire collected information on age, gender, and years of education. A measure of general negative affect; The Depression Anxiety and Stress Scale (DASS-21^86^) was administered as a screening tool. Exclusion criteria included previous diagnoses of a neurological disorder or inability to speak or understand English with basic competence. No participant was excluded on these bases in the current study. The demographic information of the sample is summarized in Table 6.

**Table 6:** Means and Standard Deviations of Characteristics of Younger and Older Adults.

	Younger adults N = 45		Older adults N = 22	
	M	SD	M	SD
Age (years)	19.84	2.01	68.00	8.05
Gender (M/F)	6/39		8/14	
Education (Years)	7.78	1.09	10.45	2.84
Depression Score (DASS-21)	2.84	2.67	2.50	2.90
Anxiety Score (DASS-21)	3.22	3.13	1.91	2.37
Stress Score (DASS-21)	5.82	3.83	3.96	2.56

Note: Years of formal education was recorded from the first year of secondary school onwards, i.e., completion of secondary school was recorded as 6 years of completed formal education. Gender is reported as N.

This study was carried out in accordance with the recommendations of the National Statement on Ethical Conduct in Human Research, La Trobe University Human Ethics Committee (UHEC), with written informed consent from all subjects. All participants gave written informed consent in accordance with the Declaration of Helsinki. The protocol was approved by La Trobe University Human Ethics Committee, approval number S15/19.

### Materials

#### Oculomotor functions using Gazepoint eye tracker

During the RAN and text-passage tasks (described below), eye movements were recorded using the Gazepoint (www.gazept.com) GP3 60 Hz gaze tracker which provided data on number of fixations, fixation duration, saccade duration in milliseconds per stimulus and per trial. This data was recorded on the Open Gaze Application Programming Interface on the Gazepoint analysis software. The Gazepoint tracker tracks vertical and horizontal eye positions with an average gaze position accuracy of 0.5°. Prior to each trial, participants’ eye gaze was calibrated using the 9-point calibration.

#### Rapid Automatized Naming (RAN) task

**Figure 3:**
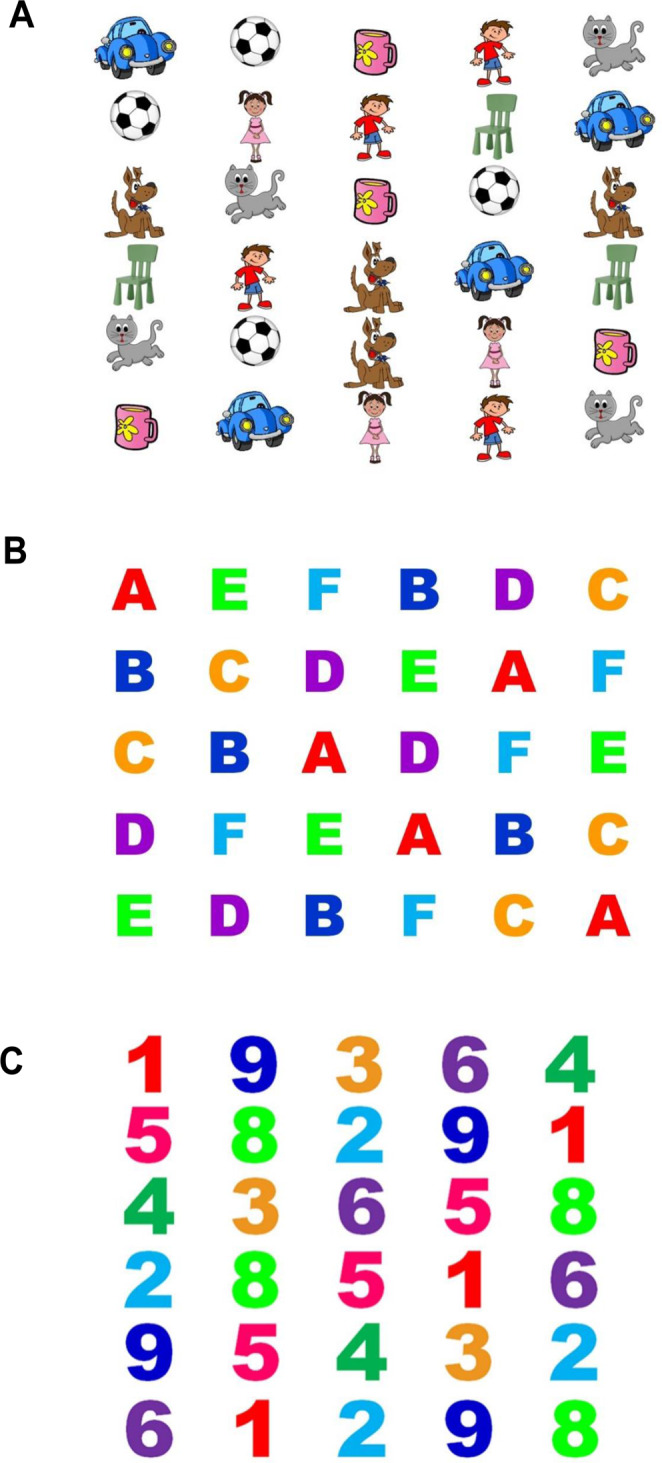
(**A**) Rapid Automatized Naming (RAN) task – Objects Condition. (**B**) Rapid Automatized Naming (RAN) task – Letters Condition. (**C**) Rapid Automatized Naming (RAN) task – Numbers condition.

The RAN tests were administered on a 24-inch Dell computer monitor. Participants were seated approximately 57 cm away from the screen and used a chin rest to maintain position and minimize head movements. The RAN comprised three rapid naming tasks (i.e., numeric, alphabetic, and common objects) that included 30 items that were presented as a single-screen array of 6 rows of 5 items for the numbers and objects, and 5 rows of 6 items for the letters condition. The visual angle of each object/letter/number in the RAN task was 2 × 2°. The RAN stimuli as depicted in Fig. 3 were developed in our lab and have been used in other research from our lab^51,87^. In the numeric condition, participants were presented with single-syllable colored numbers ranging from 1–9 presented in Arial Black font, in 72 pt, and participants were required to sequentially name each number out loud from left to right to the bottom of the page, as fast and accurately as possible. Once they reached the last number on the page, they were required to continue naming the numbers until the task ceased after 60 seconds.

In the alphabetic condition, participants were presented with single-syllable colored letters ranging from A-Z and given the same instructions as in the numeric condition. In the common objects condition, participants were presented with single-syllable cartoon images on a screen which comprised a *car, ball, cup, boy, girl, cat* and *chair*, presented in varying order across rows. Participants were given the same instructions as in the alphanumeric conditions. To ensure consistency, researchers practiced the *correct* names of each object with participants, to ensure participants used the same names for objects across trials. The quantity of numbers, letters, and objects named in 60 seconds was recorded manually and via *Gazepoint*, with higher scores indicative of a faster rapid automatized naming speed. See Fig. 3A–C for examples of the task stimuli.

### Text-passage task

**Figure 4:**
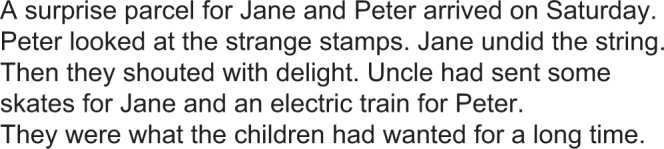
Text-Passage task.

The text-passage task was administered via computer on a 24-inch Dell monitor and assessed word-and passage-reading fluency. The text-reading task consisted of a short 49-word passage presented in 18 pt Calibri font in narrative order. Participants were instructed to read the passage once, as quickly and as accurately as possible. Time taken to read the full story as well as each individual word was recorded in milliseconds manually and on *Gazepoint*. See Fig. 4.

**Figure 5:**
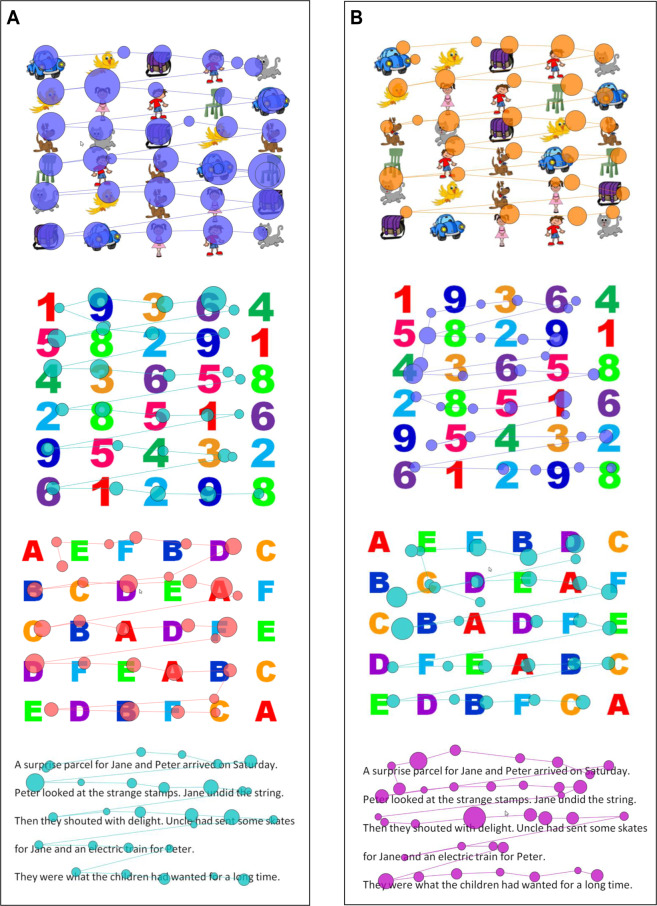
(**A**) Example of scan path for each task whereby eye-tracking was utilized. Circles represent fixation points, with larger circles indicating longer fixation durations. This example was derived from a young adult participant (19-year-old female). Please note, for the purpose of clear visualization of scan paths in this figure, the RAN tasks only show one round of the trial and not the full 60 second trial. (**B**) Example of scan path for each task whereby eye-tracking was utilized. Circles represent fixation points, with larger circles indicating longer fixation durations. This example was derived from an older adult participant (65-year-old-female). Please note, for the purpose of clear visualization of scan paths in this figure, the RAN tasks only show one round of the trial and not the full 60 second trial.

An example of scan paths for each task is provided in Fig. 5 for younger and older adults.

### Inspection Time (IT)

**Figure 6:**
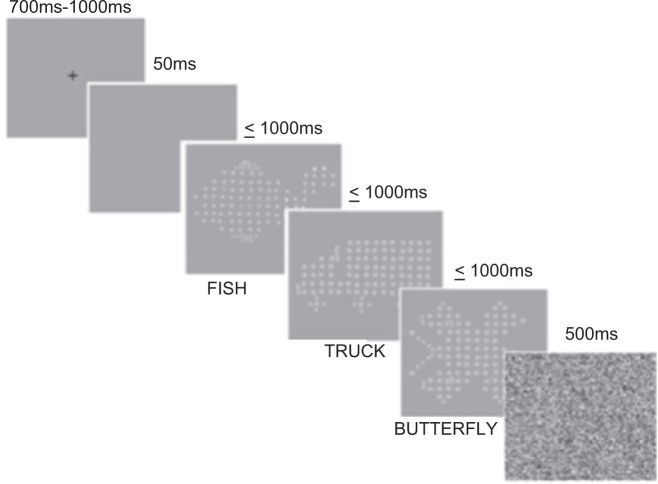
Modified Inspection Time (IT) task trial. *Note*. Only one target stimuli of fish, truck, or butterfly is presented per trial. Figure is unmodified from “Visual Information Processing in Young and Older Adults”, by D. Ebaid and S.G. Crewther^19^, Frontiers in Aging Neuroscience, 11, Copyright, 2019. Licensed under CC BY 4.0.

A modified Inspection Time (IT) task, based on the version of Vickers^52^ was adapted using Vpixx (www.vpixx.com) by Brown and Crewther^88^ and was used as a non-motor visually driven cognitive task. This task was also utilized in a previous study conducted by our lab^19^, and so this methodology has also been described in our previous research^13,19^. The task employs an inbuilt Visual Parameter Estimation by Sequential Testing (VPEST) algorithm, designed to estimate the minimum exposure threshold required to discriminate and identify which of the three possible stimuli consisting of either a *fish, truck* or *butterfly* was presented (See Fig. 6). The task was presented at suprathreshold contrast on an Apple iMac (Retina 4 K) computer with a 21.5-inch monitor running at 60 Hz screen refresh rate. Participants were required to identify the target stimulus from the three options (fish, truck or butterfly) by manually responding on a keyboard after the stimulus had disappeared. Prior to each trial, a fixation cross was presented for a random duration between 700–1000 ms, followed by a blank screen for 50 ms, after which the target stimulus was presented for variable exposure times for no greater than 1000 ms. Presentation of target stimulus was immediately followed by a mask, presented for 500 ms. The start of the next trial was triggered 100 ms after the termination of the mask. Confidence intervals and estimations of exposure time were calculated as part of the Vpixx program, where the lowest reoccurring exposure time is used to estimate and indicate the threshold duration of each individual’s perceptual time required to discriminate and accurately identify visual stimuli. See Fig. 6 for an example of the task.

### Change Detection (CD)

**Figure 7:**
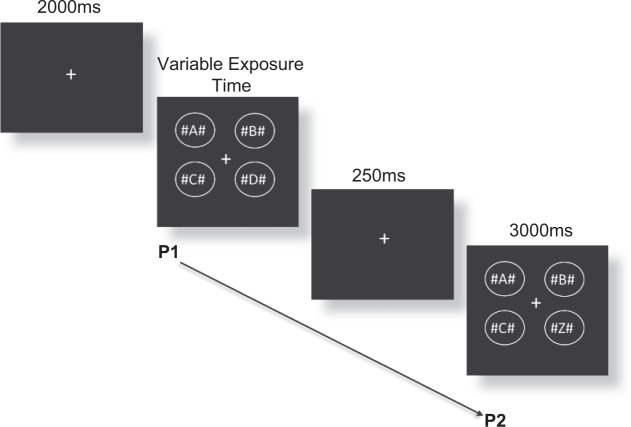
Modified Change Detection (CD) task trial where a change has occurred from presentation 1 to presentation 2. Figure is unmodified from “Visual Information Processing in Young and Older Adults”, by D. Ebaid and S.G. Crewther^19^, Frontiers in Aging Neuroscience, 11, Copyright, 2019. Licensed under CC BY 4.0.

The Change Detection (CD) task was a visual cognitive non-motor measure based on the Becker, Pashler^89^ version, as adapted by Rutkowski, Crewther^53^ and utilized the same software and VPEST technique as the IT task. This task was also utilized in a previous study conducted by our lab^19^, and so this methodology has also been described in our previous research^19^. The stimuli were four different alphabetic letters with a hash (#) symbol on either side of each letter, contained in a circle, presented on an Apple iMac (Retina 4 K) computer with a 21.5-inch monitor running at 60 Hz screen refresh rate. The four circles were arranged into a square shape (See Fig. 7). The addition of hash symbols around each letter was included to create visual crowding^90^ and used as non-alphabetic task-irrelevant stimuli, as the 4 alphabetic letters alone in a single array are well within the limit of visual-short term memory capacity^91^. A fixation cross was presented for 2 seconds at the start of each trial, followed by the first stimulus array of four letters for variable exposure times, and immediately followed by a 250 ms delay, and then another array of four letters (presented for a period of 3000 ms). The conditions which were presented in random order were a *change* condition, whereby one of the four letters were changed in the final presentation, and a *no-change* condition, where the exact same letters were shown in both presentations. Participants were required to indicate whether there was a *change* or *no-change* after each set of visual arrays, and the estimated the minimum exposure time for detection of change between the two arrays was calculated. Confidence intervals and threshold estimations of exposure time were calculated by Vpixx, where a lower estimated exposure time indicated a faster threshold response time needed to detect change between visual stimuli. An example of the task is shown at Fig. 7.

### Procedure

Testing took place in a quiet room either at La Trobe University, Melbourne, or at U3A Manningham where only the participant and experimenter were present. All participants were guided through the experimental tasks and the order of administration of tasks were counterbalanced between participants. Total testing time took approximately 30 minutes.

### Data analysis and screening

Variables collected included threshold exposure duration (ms) required to identify a visual stimulus during the IT task, and threshold exposure duration required to detect change between two visual arrays on the CD task. If confidence intervals were below 80% for the threshold estimations, the tasks were redone by participants. For the RAN tasks, naming score was collected, which represented the number of stimuli named by participants in 60 seconds per condition. For the reading task, reading score represented the duration (ms) participants took to read each word in the passage. Preliminary analysis of age-group differences in articulatory speed was conducted using an independent samples t-test to ensure that articulatory speed between age-groups was not contributing to age-group differences on the RAN tasks. Articulatory speed was measured using the *FastaReada*, which is a customized computer-generated task^55,92^, designed to measure reading fluency by determining the number of words an individual can accurately read per minute. Results demonstrated no significant differences on *FastaReada* scores between age-groups, (*t* (65) = 1.899, *p* = .346, *η*^*2*^ = 009). Oculomotor measures were collected while participants completed the RAN and reading tasks. Oculomotor variables included number of visual fixations, fixation duration (ms), and saccade duration (ms) from one fixation point to the next, all collected during the RAN and reading tasks. All analyses were performed using SPSS v. 25.0 (IBM Corp., Armonk, NY, USA). Data was screened for outliers on individual tasks and for any result indicating inconsistent performance across the different task conditions. None of these cases were found in the dataset.

## Data Availability

The datasets generated during and/or analysed during the current study are available from the corresponding author on reasonable request.
